# Supplementary material for: Supplementation with >Your< Iron Syrup Corrects Iron Status in a Mouse Model of Diet-Induced Iron Deficiency
Source: Biology (Basel). 2021 Apr 22;10(5):357. doi: 10.3390/biology10050357 (PMC8147123; doi:10.3390/biology10050357)
Supplement: Supplementary file 1 [file biology-10-00357-s001.zip › biology-1168061-supplementary.pdf]

SUPPLEMENT

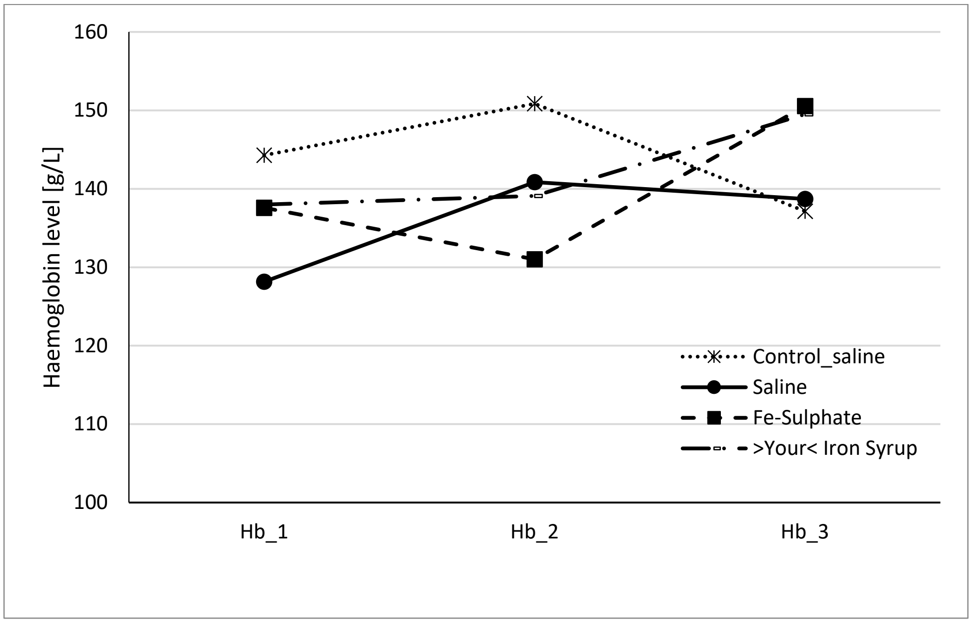

Figure S1. Haemoglobin concentration of female mice at the beginning of the gavage (Hb_1), after one week (Hb_2) of the gavage treatment and at the end of two-week gavage treatment (Hb_3).

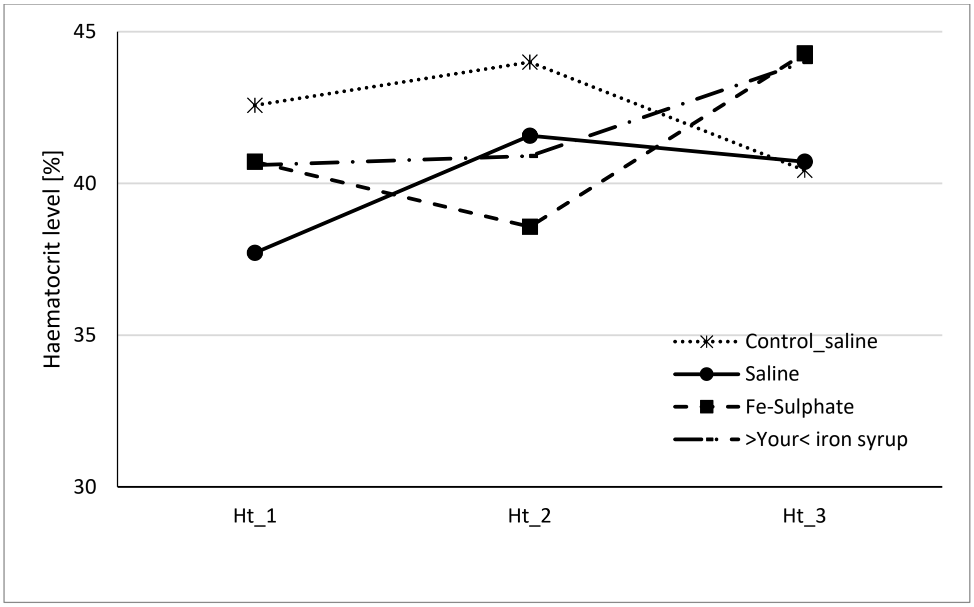

Figure S2. Haematocrit level of female mice at the beginning of the gavage (Ht_1), after one week (Ht_2) of the gavage treatment and at the end of two-week gavage treatment (Ht_3).

Table S1. Body mass and consumed diet (females) (average ± SEM). Differences between groups were not significant (p>0.05).

	Initial body mass (g)	Final body mass (g)	Consumed diet (g/day)
Control_saline	23.73 ± 0.52	21.73 ± 0.42	3.37 ± 0.17
Iron deficient diet groups			
Saline	22.87 ± 0.52	21.82 ± 0.42	3.11 ± 0.17
Fe-sulphate	22.28 ± 0.52	21.41 ± 0.42	3.13 ± 0.17
>Your< Iron Syrup	22.13 ± 0.43	21.38 ± 0.35	3.22 ± 0.15

Table S2. Body mass and consumed diet (males) (average ± SEM). Differences between groups were not significant (p>0.05).

	Initial body mass (g)	Final body mass (g)	Consumed diet (g/day)
Control_saline	27.77 ± 0.64	27.95 ± 0.68	3.62 ± 0.16
Iron deficient diet groups			
Saline	26.39 ± 0.64	25.91 ± 0.68	3.52 ± 0.16
Fe-sulphate	27.24 ± 0.60	27.02 ± 0.64	3.49 ± 0.16
>Your< Iron Syrup	30.39 ± 0.53	28.85 ± 0.57	3.65 ± 0.14

Table S3. Liver and spleen mass in males and females (average ± SEM). Differences between groups were not significant (p>0.05).

	Liver mass (g)		Spleen mass (mg)	
	Male	Female	Male	Female
Control_saline	1.15 ± 0.05	0.90 ± 0.03	132.5 ± 1.2	127.7 ± 0.7
Iron deficient diet groups				
Saline	1.10 ± 0.05	0.80 ± 0.03	118.7 ± 1.2	102.4 ± 0.7
Fe-sulphate	1.08 ± 0.05	0.82 ± 0.03	104.3 ± 1.1	115.3 ± 0.7
>Your< Iron Syrup	1.24 ± 0.04	0.83 ± 0.02	111.4 ± 1.0	103.0 ± 0.6

Table S4. Haemoglobin concentration (g/L) (average ± SEM) before and after two weeks of gavage feeding. Differences between groups were not significant (p>0.05).

	Male		Female	
	before	after	before	after
Control_saline	144.3 ± 6.7	137.4 ± 7.8	144.3 ± 4.5	137.1 ± 4.5
Iron deficient diet groups				
Saline	115.4 ± 6.7	108.4 ± 7.8	128.1 ± 4.5	138.7 ± 4.5
Fe-sulphate	127.6 ± 6.3	138.6 ± 7.3	137.6 ± 4.5	150.6 ± 4.5
>Your< Iron Syrup	121.6 ± 5.6	139.3 ± 6.5	138.0 ± 3.8	149.5 ± 3.7

Table S5. Haematocrit level (%) (average ± SEM) before and after two weeks of gavage feeding. Differences between groups were not significant (p>0.05).

	Male		Female	
	before	after	before	after
Control_saline	42.0 ± 2.0	40.6 ± 2.3	42.6 ± 1.4	40.4 ± 1.3
Iron deficient diet groups				
Saline	32.2 ± 2.0	31.8 ± 2.3	37.7 ± 1.4	40.7 ± 1.3
Fe-sulphate	35.3 ± 1.8	39.3 ± 2.2	40.7 ± 1.4	44.3 ± 1.3
>Your< Iron Syrup	35.6 ± 1.6	40.9 ± 1.9	40.6 ± 1.1	44.0 ± 1.1

Table S6. qPCR expression analyses of four inflammatory genes (*Crp*, *Saa1*, *Il6* and *Soc3*) in the liver (average ± SEM).

	Crp (dC_T)		*Saa1* (dC_T)	
	Male	Female	Male	Female
Control_saline	2.59 ± 0.43	1.26 ± 0.51	-2.32 ± 1.07 [b]	4.13 ± 0.65
Iron deficient diet groups				
Saline	2.86 ± 0.43	1.65 ± 0.67	3.04 ± 1.07 [a]	5.05 ± 0.65
Fe-sulphate	2.76 ± 0.56	1.64 ± 0.48	-1.36 ± 1.38 [ab]	4.66 ± 0.75
>Your< Iron Syrup	2.98 ± 0.43	1.62 ± 0.51	2.23 ± 1.07 [ab]	3.56 ± 0.46
	Il6 (dC_T)		*Soc3* (dC_T)	
	Male	Female	Male	Female
Control_saline	10.06 ± 0.75 [ab]	10.01 ± 0.94	6.85 ± 0.50	7.02 ± 0.42
Iron deficient diet groups				
Saline	11.13 ± 0.75 [ab]	11.89 ± 0.94	6.93 ± 0.50	7.74 ± 0.56
Fe-sulphate	7.94 ± 0.96 [b]	11.41 ± 0.94	7.60 ± 0.65	7.05 ± 0.56
>Your< Iron Syrup	12.68 ± 0.84 [a]	10.23 ± 0.67	6.33 ± 0.50	6.18 ± 0.40

[ab] Columns without the same superscript differ significantly between the groups inside the sex ($p \leq 0.05$).

p- value for the sex effect: *Crp*: p=0.0030; *Saa1*: p<0.0001
